# Prospective multicentre study of the effect of voluntary plasmapheresis on plasma cholesterol levels in donors

**DOI:** 10.1111/vox.12031

**Published:** 2013-03-20

**Authors:** M Rosa-Bray, C Wisdom, S Wada, BR Johnson, V Grifols-Roura, V Grifols-Lucas

**Affiliations:** 1 Grifols, Plasma OperationsLos Angeles, CA, USA; 2 CW Clinical Regulatory Consulting, IncPasadena, CA, USA; 3 WestatRockville, MD, USA; 4 Grifols S.ABarcelona, Spain

**Keywords:** cholesterol level, plasma donation, plasma donor, plasmapheresis

## Abstract

**Background and Objectives** LDL apheresis is used to treat patients with familial hypercholesterolaemia, and low-volume plasmapheresis for plasma donation may similarly lower cholesterol levels in some donors. This study was designed to assess the effect of plasmapheresis on total, LDL and HDL cholesterol levels in a plasma donor population.

**Materials and Methods** This was a prospective, unblinded longitudinal cohort study in which a blood sample was obtained for analysis before each donation. Data from 663 donors were analysed using a multivariable repeated measures regression model with a general estimating equations approach with changes in cholesterol as the primary outcome measure.

**Results** The model predicted a significant decrease in total and LDL cholesterol for both genders and all baseline cholesterol levels (*P* < 0·01). The greatest total cholesterol decreases (women, −46·8 mg/dL; men, −32·2 mg/dL) were associated with high baseline levels and 2–4 days between donations. Small but statistically significant increases (*P* ≤ 0·01) in HDL cholesterol were predicted for donors with low baseline levels.

**Conclusions** These results suggest that, in donors with elevated baseline cholesterol levels, total and LDL cholesterol levels may decrease during routine voluntary plasmapheresis.

## Introduction

Plasma products are therapeutic agents purified from human plasma. Plasma is collected by automated plasmapheresis and involves removal of blood from the donor, separation of plasma and return of cellular components to the donor 1–2. The plasma is processed to extract and purify its components into biological therapeutics. Plasma collection and the manufacturing of plasma products are conducted under rigorous standards set by national and international regulatory agencies.

Plasmapheresis in a healthy donor population was first described in 1952 3. Since then, the plasma products industry has carefully monitored donor safety and the effects of repeated plasma donations on long-term donors. Industry experience involving over 400 licensed donation centres and over 20 million donations per year 4 has demonstrated that plasmapheresis is generally a safe process, with the most common adverse event being a mild vasovagal response. Studies continue to confirm that donors undergoing plasmapheresis under regulated parameters do not report significant donation-related adverse events 5–8. Donors generally experience a low incidence of adverse events 6, and most dropouts are related to nonmedical reasons or medical reasons unrelated to plasma donation 7,8. This parallels findings in studies with blood donors. Studies of plasma proteins have generally shown that the levels of total serum protein, immunoglobulin, albumin and globulin, and possible iron storage, are lower in experienced donors undergoing frequent plasmapheresis than in controls 7–13. However, in one study of donors who donated regularly for at least 12 months prior to enrolment, there were no changes in total (TC), low-density lipoprotein (LDL) and high-density lipoprotein (HDL) cholesterol regardless of donation frequency 7–11.

Therapeutic LDL apheresis is approved for treatment of refractory hypercholesterolaemia. Although frequent treatments are required, this therapy can lower plasma LDL and may result in regression of atherosclerotic plaque and decreased frequency of cardiovascular events 14–18 with an immediate 76%–81% reduction in LDL cholesterol observed following apheresis in one study 18. However, apheresis is not used as first-line therapy as there are few medical centres equipped to provide the treatment, and it is not favoured by patients due to the lengthy procedure time and the need for two venipunctures.

There are important differences between therapeutic apheresis and nontherapeutic donor plasmapheresis. In therapeutic apheresis, the volume of plasma mobilized and the volume of sodium citrate administered are higher, and treatment-related adverse events are frequently reported 19. In contrast, plasmapheresis for plasma donation involves a single venipuncture, takes at most 1 h and involves smaller volumes of plasma (<800 ml) and sodium citrate (<100 ml). Compared to blood donation, although both processes involve plasma removal, blood donation also involves the removal of cellular components 6–20. As the beneficial effects of therapeutic apheresis are well known for patients with familial hypercholesterolaemia, it seemed likely that plasmapheresis of healthy plasma donors might lower cholesterol levels in some donor populations, especially those with high baseline cholesterol.

## Materials and methods

### Study design

This was a prospective longitudinal cohort study conducted with institutional review board approval (Westat, Rockville, MD) at nine plasma donation centres throughout the United States. Potential study participants were either new plasmapheresis donors or previous donors who had not donated for at least 6 months.

All participants were required to meet eligibility criteria for plasma donation and to sign both the routine consent to donate plasma and a separate study consent. Participants were between 18 and 69 years of age and met all routine plasma industry standards for weight (≥110 pounds), blood pressure, pulse, temperature, haematocrit and total protein, passed a physical examination and provided a detailed medical history. Participants were required to complete two donations during the recruitment period and to have negative serological and nucleic acid testing (NAT) for HIV, hepatitis B and hepatitis C, negative hepatitis A (NAT), parvovirus B19 (NAT) and rapid plasma reagin, and normal baseline serum protein electrophoresis, alanine amino transferase (ALT), urine glucose and urine protein. All individuals accepted as plasma donors were invited to participate in the trial.

Study participants were asked to donate at least once weekly for 16 weeks. However, the plasma donation frequency permitted by the FDA (twice in 7 days) was allowed, for a possible maximum of 32 study donations. Prior to each donation, study participants were required to meet routine eligibility criteria including vital signs, haematocrit, total protein, weight and weight changes, and medical, social and travel criteria. Participants also completed a questionnaire to monitor changes in diet, physical activity or the use of medications that could impact cholesterol levels.

### Donation procedure

Plasmapheresis procedures were identical to standard donor centre procedures with the exception that a 5-ml nonfasting blood sample was obtained just prior to the initiation of plasmapheresis. Following US guidelines, the volume of plasma collected (690, 825 or 880 ml after citrate) was determined by the donor’s weight. Study participants were provided with their cholesterol test results every 2 months. Donors with high or borderline TC or LDL in 50% or more samples could have a fasting blood sample analysed if they wished to provide these results to their personal physician.

### Laboratory testing

Cholesterol testing was performed at the Grifols Plasma Testing Laboratory in Austin, Texas, using an Abbott Architect c8000 analyzer and Architect/Aeroset reagents. As samples were collected when donors were nonfasting, direct LDL was measured using the Abbott Architect c8000 analyzer with the Multigent – Direct LDL reagent. Triglyceride was measured, but data were not evaluated as the nonfasting state affects triglyceride levels.

### Adverse reactions and donor deferrals

Adverse reactions occurring during or following donation, including events occurring after donors left the centre, were documented as per company procedures. The company’s internal classification system for donor reactions, based on the combination of symptoms and the recovery period, was used. Reactions were classified as mild, moderate or severe.

Study participants failing to meet donation screening criteria at each visit were temporarily deferred, but remained in the study and could donate following the expiration of their deferral period if they met all criteria. Most temporary deferrals were 1-day deferrals, although the donor often returned at a later date. Donors could be permanently deferred for administrative reasons, positive test result for an infectious disease or if recommended by the personal or centre physician. Donors who received permanent deferrals could not donate plasma or participate in the study, although data obtained prior to deferral remained in the data set.

### Statistical analysis

Data were analysed using a multivariable repeated measures regression model with a general estimating equations (GEE) approach 21–22, which accounted for the likely correlation of cholesterol within individual donors. Also, it accounted for unbalanced data, that is, varying numbers of follow-up visits and differing times between donations, which could be problematic for other statistical methodologies. This type of model appropriately handles data that are clustered by individual and may be interpreted in much the same manner as a multivariable regression model.

Variables considered to be possible predictors for change in cholesterol levels included gender, age, weight (and therefore donation volume), race, baseline cholesterol levels, total number of donations, number of donations prior to a measurement and days between donations. These variables were added and removed in a stepwise procedure, testing the overall effect and statistical significance of each predictor. The variables baseline cholesterol level, time between donations and gender were selected as predictors in the final model as the others showed no significant effect on cholesterol-level change. Each predictor interacted with the others, and the final model included a 3-way interaction of these variables, with donations classified into 18 groups based on the various combinations of baseline cholesterol level (3 categories), gender (2 categories) and days between donations (3 categories). Baseline cholesterol levels were grouped according to the recommendations of the National Cholesterol Education Program 23. Donation interval categories were 2–4 days, 5–9 days and 10 or more days.

All descriptive frequencies and modelling were performed using sas 9.2 (SAS Institute, Inc., Cary, NC).

## Results

A total of 666 donors were enrolled; data from 663 donors were analysed. Two donors who completed only one donation and one donor who took cholesterol-lowering medications were excluded from analysis. Overall, 61% of donors were men (Table 1). Both male and female donors had similar profiles, with the typical donor being in the youngest age group, of white race and weighing <200 pounds. Many (44·6%) participants gave between two and 10 donations; however, 199 participants (30·0%) donated 21–32 times.

**Table 1:** Demographic and donation characteristics stratified by gender

		Females, *n* (%) (N = 256)	Males, *n* (%) (N = 407)
Age (yr)	18–24	99 (38·7)	164 (40·3)
	25–34	81 (31·6)	137 (33·7)
	35–44	44 (17·2)	59 (14·5)
	≥45	32 (12·5)	47 (11·5)
Race/Ethnicity	White	112 (43·7)	192 (47·2)
	African American	33 (12·9)	55 (13·5)
	Hispanic	45 (17·6)	74 (18·2)
	Not available	66 (25·8)	86 (21·1)
Weight (lbs)	<200	174 (68·0)	253 (62·2)
	200–249	60 (23·4)	104 (25·5)
	250–299	14 (5·5)	35 (8·6)
	≥300	8 (3·1)	15 (3·7)
Total study donations	2–10	136 (53·1)	160 (39·3)
	11–20	66 (25·8)	102 (25·1)
	21–32	54 (21·1)	145 (35·6)

Over 72% of donors had acceptable baseline TC (Table 2), while <6% had high TC. Mean (SD) cholesterol levels of 180·6 (36·8) mg/dL were observed in female donors and 176·8 (36·8) mg/dL in men. Over 76% of participants had acceptable baseline LDL; mean LDL levels were 109·5 (30·6) mg/dL and 108·0 (31·2) mg/dL in men and women, respectively. Male donors had mean HDL levels of 47·1 (10·9) mg/dL compared to 51·9 (14·0) mg/dL in women. Most male donors (61·7%) but only 35·1% of female donors presented with average baseline HDL.

**Table 2:** Baseline cholesterol levels (mg/dL) stratified by gender

		Females, *n* (%) (N = 256)	Males, *n* (%) (N = 407)
Total Cholesterol (mg/dL)	High (≥240)	14 (5·5)	24 (5·9)
	Higher than desired (200–239)	57 (22·3)	75 (18·4)
	Acceptable (<200)	185 (72·3)	308 (75·7)
LDL Cholesterol (mg/dL)	High (≥160)	16 (6·2)	25 (6·1)
	Higher than desired (13–159)	44 (17·2)	68 (16·7)
	Acceptable (<130)	196 (76·6)	314 (77·2)
HDL Cholesterol (mg/dL)	Low (<40, males, <50, females)	120 (46·9)	108 (26·5)
	Average (4–60, males, 5–60, females)	90 (35·1)	251 (61·7)
	Optimal (>60)	46 (18·0)	48 (11·8)

### Longitudinal analysis

**Figure 1:**
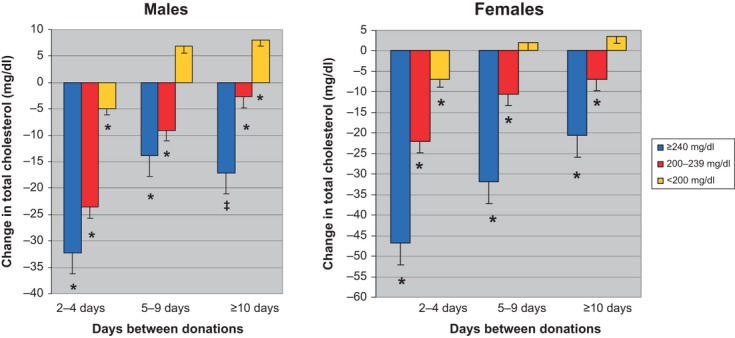
Effect of plasmapheresis on change from baseline in total cholesterol (mg/dL) in male and female donors * *P* < 0·01 ‡ *P* < 0·05.

A total of 9153 records were analysed. The predicted change from baseline in TC is shown in Fig. 1. With a 2-to 4-day donation interval, a significant (*P* ≤ 0·01) decrease in TC was predicted for both genders and all baseline levels. However, the decrease was much greater for individuals with high or higher than desired starting TC levels (−46·8 and −22·0 mg/dL for women; −32·2 and −23·6 mg/dL for men; *P* ≤ 0·01). A similar pattern for donors with high or higher than desired baseline TC was observed when donations were separated by 5–9 days; predicted levels for high baseline donors remained lower following donation intervals of 10 or more days (*P* ≤ 0·05).

**Figure 2:**
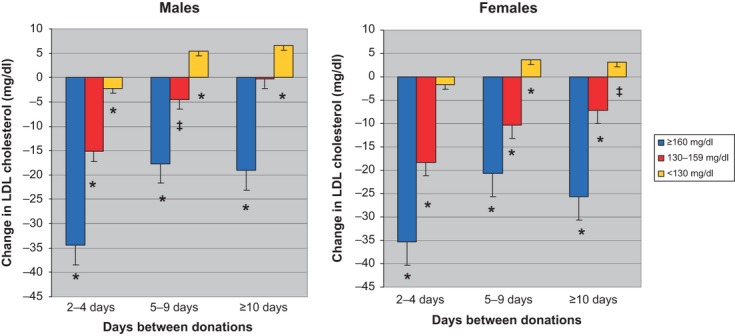
Effect of plasmapheresis on change from baseline in LDL cholesterol (mg/dL) in male and female donors * *P* < 0·01 ‡ *P* < 0·05.

Similar results were observed for the LDL model (Fig. 2) with the greatest predicted drops in LDL observed in donors with high baseline LDL (*P* ≤ 0·01 for all groups). Male donors with higher than desired starting LDL had significant decreases in cholesterol with 2–4 days (15·2 mg/dL, *P* ≤ 0·01) and 5–9 days (4·5 mg/dL, *P* ≤ 0·05) between donations with similar changes predicted for female donors (*P* ≤ 0·01). For donors with acceptable starting LDL, the predicted magnitude of change tended to be small and without a clear pattern.

**Figure 3:**
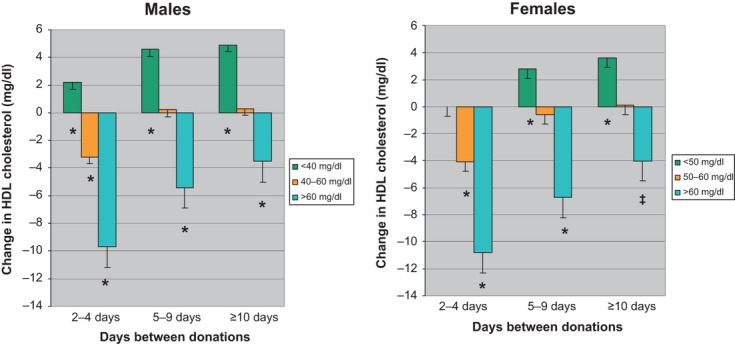
Effect of plasmapheresis on change from baseline in HDL cholesterol (mg/dL) in male and female donors * *P* < 0·01 ‡ *P* < 0·05.

For HDL, where higher levels are considered positive, a small but statistically significant increase was predicted for donors with a low baseline HDL (Fig. 3). For donors with optimal HDL, small but statistically significant decreases in donor HDL were predicted for 2–4 or 5–9 (*P* ≤ 0·01) and 10 or more (*P* ≤ 0·05) days between donations. HDL levels remained within the optimal or acceptable ranges for these donors.

To evaluate the fit of each model, the observed and predicted values of cholesterol as it differed from baseline were compared (Table 3). This comparison confirms that the models fit the data reasonably well and explain much of the observed variation in cholesterol.

**Table 3:** Differences between predicted and observed changes in total, LDL and HDL cholesterol (mg/dL) following plasmapheresis

Gender	Days between donation	Observed difference/discrepancy with predicted difference		
Total cholesterol (Baseline) (days)		High (≥240 mg/dL)	Higher than desired (200–239 mg/dL)	Acceptable (<200 mg/dL)
Female	2–4	−44·8/−2	−22·2/0·2	−4·5/−2·5
	5–9	−29·2/−2·6	−10·7/0·2	3·2/1·1
	≥10	−19·6/−0·9	−7·9/1	1·6/−2
Male	2–4	−30·0/−2·2	−23·6/0	−2·0/−2·9
	5–9	−10·7/−3·1	−8·8/−0·2	9·3/2·5
	≥10	−22·7/5·6	−3·7/1	7·2/−0·8
LDL cholesterol (Baseline) (days)		High (≥160 mg/dL)	Higher than desired (130–159 mg/dL)	Acceptable (<130 mg/dL)
Female	2–4	−32·6/−2·8	−15·9/−2·4	0·2/−1·4
	5–9	−17·8/−2·9	−8·2/−2·1	4·3/0·6
	≥10	−19·2/−6·5	−10·7/3·5	2·0/−1·2
Male	2–4	−33·8/−0·6	−15·4/0·2	−0·3/−2
	5–9	−16·6/−1·1	−4·4/−0·1	7·3/1·9
	≥10	−20·4/1·3	−6·1/5·8	6·4/−0·2
HDL cholesterol (Baseline) (days)		Low (<40 mg/dL male, <50 mg/dL female)	Near optimal (40–60 mg/dL male, 50–60 mg/dL female)	Optimal (>60 mg/dL)
Female	2–4	0·3/0·3	−4·2/0·1	−12·6/1·8
	5–9	2·8/0	−0·8/0·2	−7·2/0·5
	≥ −10	2·2/−1·4	−0·2/0·1	−3·7/−0·3
Males	2–4	2·7/0·5	−3·2/0	−10·6/0·9
	5–9	5·0/0·4	0·2/0	−6·0/0·6
	≥10	4·5/−0·4	0·1/−0·2	−2·7/−0·8

### Donor safety

A total of 14 adverse events (8, mild; 6, moderate) were reported in 14 donors (10 men; 4 women). Three events occurred during the initial donation, and an additional 7 occurred during the second or third donation; only four events were reported after five or more donations. Donors reporting AEs generally (92·8%) had normal baseline cholesterol levels.

Ten adverse events presented with more than one symptom, with vasovagal events being the most common events. The most frequently observed symptoms were dizziness (8 instances), nausea (7 instances) and loss of consciousness (6 instances). Blurred vision, pallor, vomiting and haematoma were also reported. A single report of chest pain was described as tightness in the chest. This event was self-limited, resolved without intervention and was not related to a cardiac aetiology. There were no severe or serious donor reactions; no cellulitis or injury was reported, and no donors required hospitalization or further care by their personal physician.

Only 12 donors were permanently deferred during the study period: 9 were deferred for administrative reasons, 2 for viral marker positivity and one for elevated ALT. A total of 484 donors received one or more temporary deferrals (1–13 deferrals per person); donors were most commonly deferred for administrative reasons unrelated to a medical condition. The most common temporary medical deferrals were a 1-day deferral for unacceptable results for haematocrit (118 donors, 17·8%), temperature (59 donors, 8·9%) and pulse (53 donors, 8·0%). Deferrals were not correlated with donation frequency or total number of donations.

## Discussion

This study demonstrates that, in donor populations with high or higher than desired baseline total or LDL cholesterol, a significant drop in cholesterol levels is expected when donations occur at 2-to 4-day intervals. A small decrease is still predicted when the donation interval increases. These predicted changes were confirmed by comparison with the actual changes observed in donors with high or higher than desired baseline cholesterol levels. The finding is consistent with the changes observed following LDL apheresis in patients with familial hypercholesterolaemia 15–17 and suggests that plasmapheresis in donors with high baseline cholesterol may temporarily result in substantially lower total and LDL cholesterol levels. This may result from the establishment of a new equilibrium in which the rates of cholesterol biosynthesis and removal (including removal through plasmapheresis) result in lower total and LDL cholesterol levels while plasma donations are continued.

Donors were asked to donate once weekly, but were allowed to follow FDA guidelines permitting two donations per week with at least one full day between donations. The 2-to 4-day donation interval employed in the model included donations with 1-to 3-day nondonation intervals. While a donor could have donated twice weekly using this interval range, donations may also have been made at longer intervals prior to or following these donations.

Previous studies have shown no change in cholesterol levels in the donor populations examined 7–11. However, these reports evaluated changes in ongoing long-term donors. It is possible that the baseline cholesterol levels observed in those studies did not reflect cholesterol levels on donor entry into the plasmapheresis programmes. This study employed new donors or donors who had not donated for at least 6 months and were more likely to show the effect of plasmapheresis initiation. Based on our model’s prediction, donors in previous studies are expected to have achieved a lower cholesterol equilibrium prior to entering the trial.

Previous studies also evaluated cholesterol levels for the study population as a whole, and the differential effects in a subgroup with higher cholesterol levels were not assessed. In preliminary analyses of our population, no marked effects were observed when the donor group as a whole was examined, possibly due to the preponderance of normocholesterolaemic donors. However, when donors with high baseline cholesterol were examined separately, we noted a decrease in cholesterol levels. Additionally, the model demonstrated that, although statistically significant decreases in cholesterol levels were expected with a donation interval of 2–4 days, these changes were minor in donors with normal baseline total or LDL cholesterol, strengthening the findings in the high baseline cholesterol population.

The differences observed are likely not due to day-to-day variability in serum cholesterol. Drops of 17·1–46·8 mg/dL were predicted in donors with high baseline TC, notably more than the day-to-day variability of 9 mg/dL observed for TC in another study 24. Further, as donors taking cholesterol-lowering drugs were excluded from analysis and no lifestyle changes were reported that could affect cholesterol levels, the decreases probably did not result from external factors.

When donations occurred at 2-to 4-day intervals, HDL levels remained unchanged or were slightly elevated in donors with low baseline HDL. However, a small but significant (*P* < 0·01) increase in HDL cholesterol was predicted for donors with low baseline HDL cholesterol and a donation interval of 10 days or more. The model predicted a significant (*P* < 0·05) decrease in HDL cholesterol for donors with optimal baseline HDL. Although this drop was markedly reduced with a donation interval of 10 days or greater, a slight reduction in HDL cholesterol levels was still observed, although no decrease to undesirable levels was predicted.

The finding of increased HDL levels following plasmapheresis in the low baseline HDL group is intriguing. A tentative explanation could be based on the perturbation of cholesterol homoeostasis. The body’s cholesterol comes from two sources: it is synthesized in the liver and consumed in fatty foods. LDL transports cholesterol to tissues for cellular use, whereas HDL transports surplus cholesterol from tissues to the liver for catalysis 25. With excess cholesterol intake, normally due to a fat-rich diet, LDL is found in high amounts in blood, HDL cannot cope with the surplus cholesterol, and it accumulates in blood vessels 26. After plasmapheresis, both LDL and HDL levels initially decrease. In a situation of excess cholesterol in the body, we postulate that as LDL depletion is beneficial, it does not induce any physiological response. However, if HDL levels are initially low, their further depletion may trigger a cholesterol homoeostatic mechanism for synthesizing more HDL in the liver.

Our findings of decreased cholesterol levels in donors with higher or higher than desired baseline cholesterol and of little effect in normocholesterolaemic donors are consistent with the view that frequent plasmapheresis may be safely performed in donor populations and that intensive plasmapheresis is not a risk factor for arteriosclerotic cardiovascular disease as suggested in an earlier report 27.

The adverse event rate observed in this study was low (0·15%, 14/9209 donations) and similar to the overall adverse event frequency (0·28%) reported in a study of blood and apheresis donations 6. As in the previous study, events were mild to moderate in severity and resolved rapidly. In this study, adverse events were most frequently reported during the first three donations. There was no evidence of an increase in adverse events following donation over the study period. This finding supports the historical evidence of the safety of plasmapheresis in donor populations.

The study has several limitations. First, the parameterization employed in the GEE approach may be overly simplistic. The current model is dependent on the choice of categories, and it is possible that modelling using other categories or nonlinear continuous variables would produce different results. However, the categories chosen appeared reasonable, are readily understood and have precedence in epidemiological literature.

Second, the study was relatively short and did not include a postdonation assessment period. The population employed permitted an evaluation of the initial plasmapheresis effects. However, without postdonation follow-up, the time required for cholesterol levels to return to baseline could not be accurately predicted, although in donors with long intervals between donations, the decrease in cholesterol levels was markedly reduced. There was no indication that reduced cholesterol levels were maintained for prolonged periods following cessation of donation. The short study duration also did not allow us to determine whether lower cholesterol levels were maintained in long-term, continuing donors. However, previous studies of experienced donors who likely had achieved a lower total and LDL cholesterol equilibrium at study baseline showed no change in cholesterol levels.

Third, as observed for other studies, relatively few donors (<200) completed the study. This was in part due to the inclusion of many first-time donors in the study who did not have first-hand familiarity with the donation process. Study discontinuation did not appear to be due to adverse events. Most dropouts who provided a reason for study discontinuation gave reasons that were unrelated to plasma donation or study participation.

Fourth, participants were provided with their cholesterol results 2 months into the study, presenting a possible bias if donors began lifestyle modifications after receiving this information. However, donor activities, including diet, medication and exercise, were followed using the visit questionnaire, and no changes in these activities were observed.

Finally, relatively few donors had high baseline total (38 donors) or LDL (41 donors) cholesterol. A strength of the model selected is its ability to extrapolate results from the data, with each data point of each individual providing an independent contribution to the overall prediction. Although it is possible that the relatively small number of donors with high baseline total and LDL cholesterol values might have some impact on the overall conclusions, the fact that the model predicted little to no change in donors with normal baseline cholesterol and the correlation between predicted and observed results strengthens the likelihood that the model’s prediction is physiologically relevant.

## Conclusion

This study suggests that, in donors with high baseline cholesterol levels, plasmapheresis performed at short intervals (2–4 days) may lower total and LDL cholesterol levels. Also, the results suggest that there is an increase in HDL cholesterol levels in donors with low baseline levels, when plasmapheresis is performed at intervals of 10 or more days. This effect seems to be independent of the total number of donations and appears most likely to reflect changes between donations. The time frame of this study and its design are insufficient to determine whether there is a cardiovascular benefit. Further studies are needed to confirm these results and to evaluate the possible clinical effects of these changes.
